# Recurrent Giant Pilomatrixoma of the Face: A Case Report and Review of the Literature

**DOI:** 10.1155/2012/197273

**Published:** 2012-10-18

**Authors:** Mohammed Nadershah, Ahmad Alshadwi, Andrew Salama

**Affiliations:** ^1^Department of Oral and Maxillofacial Surgery, Boston Medical Center, Boston University, 850 Harrison Avenue, Boston, MA 02118, USA; ^2^Oral and Maxillofacial Surgery Department, Boston Medical Center, Boston University, 100 East Newton street, Boston, MA 02118, USA; ^3^Boston Medical Center, Boston University, 850 Harrison Avenue, Boston, MA 02118, USA

## Abstract

Pilomatrixoma, also known as pilomatricoma, is a benign tumor that originates from the matrix of the hair root. It usually presents as a single, slow-growing subcutaneous or intradermal firm nodule with a general size of less than 3 centimeters (cm) in diameter. However, giant pilomatrixomas (more than 5 cm) have been reported infrequently. It is more common in females and usually presents during the first two decades of life (60%) as an asymptomatic, mobile, hard, elastic mass. Most of the cases are benign and affect the face. The authors report a rare case of a giant pilomatricoma of the cheek and discuss the surgical management of these lesions, histopathological findings, and review of the literature.

## 1. Introduction 

Pilomatrixoma, also known as pilomatricoma or calcifying epithelioma of Malherbe, was first described in 1880 by Malherbe and Chenantais [1]. In 1961, Forbis and Helwig proposed the term pilomatrixoma to emphasize the lesion origin, the matrix of the hair root [2]. pilomatrixoma is a benign skin neoplasm that usually presents as a single, slow-growing subcutaneous or intradermal firm nodule with a general size of less than 3 centimeters (cm) in diameter. However, giant pilomatrixomas (more than 5 cm) have been reported infrequently. It is more common in females and usually presents during the first two decades of life (60%) as an asymptomatic, mobile, hard, elastic mass. Most of the cases are benign and affect the face [3, 4]. We report a rare case of a giant pilomatricoma of the cheek and discuss the surgical management of these lesions, histopathological findings, and review of the literature.

## 2. Case Report

A 28-year-old male was referred to the department of oral and maxillofacial surgery for evaluation and management of a left facial mass. He had no other medical problems and no known food or drug allergies. At the age of 15 years, he noticed a mass on his left cheek eminence, which was excised and was told that it was a sebaceous cyst. Three years later, he had a local recurrence of the facial mass that was surgically excised again showing the same pathology. A third recurrence in the same area occurred 4 years later and was excised with the overlying skin. However, at this time the pathology specimen proved to be pilomatrixoma. The mass recurred again few years later and has been growing slowly over the past 3 years prior to presentation. 

**Figure 1:**
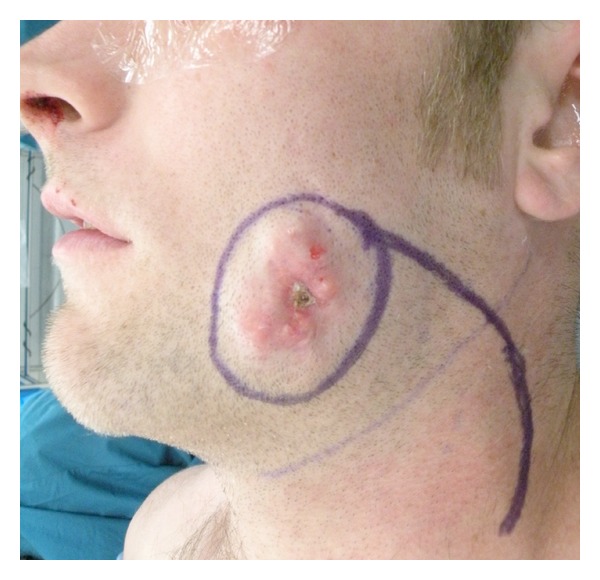
Clinical view of the lesion with calcified portion protruding from the central part of the lesion.

Clinical examination of the face showed a firm, nontender mass infiltrating the overlying skin of the left buccal subunit measuring about 5 × 3 cm. The overlaying skin had bluish discoloration (Figure 1). There was no limitation of the mandibular range of motion. Cranial nerve exam was grossly intact. The neck was supple with no palpable masses or cervical lymphadenopathy. Intraoral exam was unremarkable.

**Figure 2:**
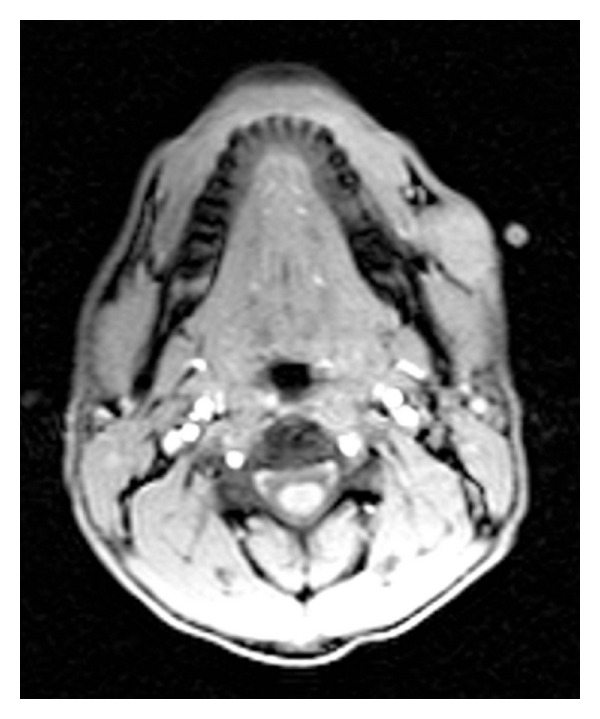
Axial view of a T2 magnetic resonance image at the level of the mandible showing a heterogeneous mass on the left side of the face with no evidence of deep invasion.

**Figure 3:**
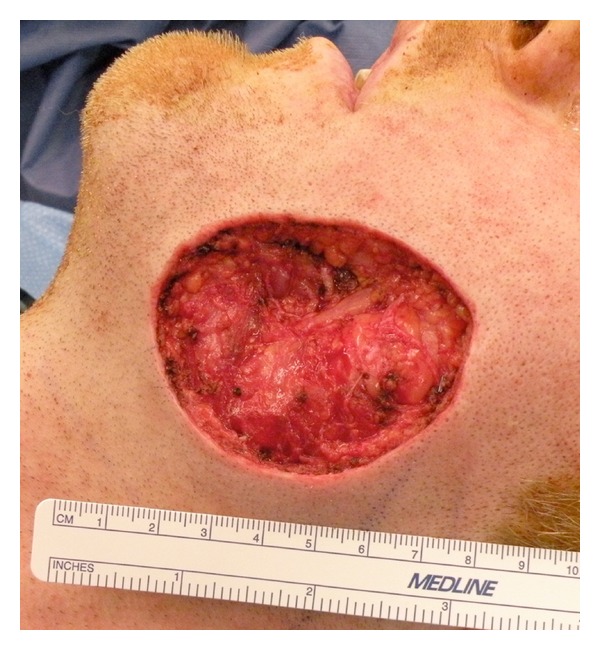
Intraoperative picture of the surgical defect measuring about 5 × 6 cm.

**Figure 4:**
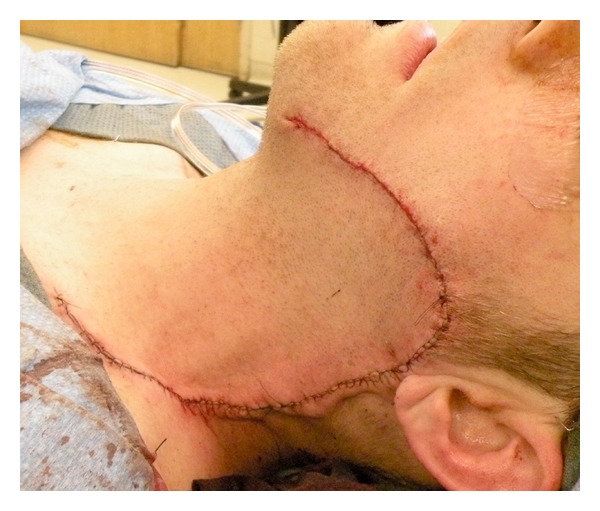
The surgical defect was reconstructed with cervicofacial advancement flap.

Another incisional biopsy was done under local anesthesia, which confirmed the diagnosis and ruled out malignant transformation. A contrast enhanced magnetic resonance imaging (MRI) study showed a heterogeneously enhancing mass in the subcutaneous tissues overlying the left platysma muscle at the level of the mandible (Figure 2). The adjacent musculature and bone marrow maintained their normal signal intensity. The mass was surgically excised including the overlying skin with a safety margin of 1 cm. About 6 × 5 cm skin was marked over the pilomatrixoma and was included in the specimen. The incision was carried through the skin, subcutaneous tissues, and the superficial musculoaponeurotic system (SMAS). The buccal and marginal mandibular branches of the left facial nerve were identified and preserved. The resulting cheek defect measured about 6 × 5 cm (Figure 3) and was reconstructed using a cervicofacial flap (Figure 4). Postoperatively, the patient recovered well without any appreciable facial nerve deficits or wound complications. He had no evidence of disease recurrence in his one-year follow up. 

## 3. Histopathologic Findings

**Figure 5:**
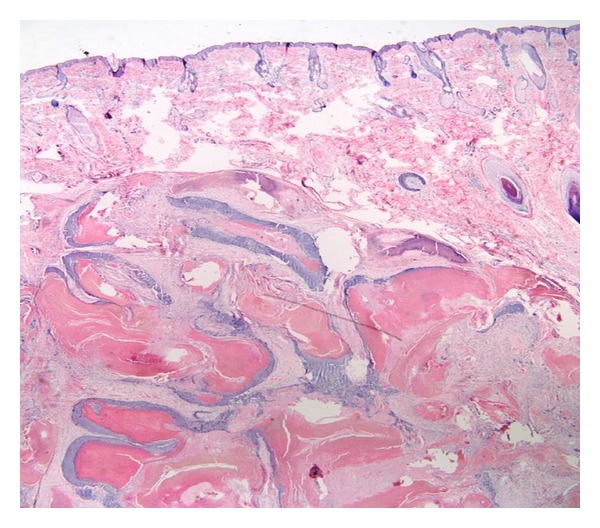
Low magnification H&E stained histopathological slide showing islands of epithelial cells with areas of calcification.

Pilomatrixomas appear as well-demarcated, lobulated lesions situated in the dermis or subcutaneous tissue. The tumor is composed of ghost cells, basaloid cells, and giant cell, in addition to keratin debris and intracellular and stromal calcifications (Figure 5) [5]. Uncommon histological features include pigmentation, transepidermal elimination, and aggressiveness with infiltrative growth pattern [6]. Malignant transformation is rare [7, 8].

## 4. Discussion 

Pilomatrixoma is an unusual neoplasm of hair germ matrix origin. Head and neck pilomatrixoma represent 50% of the reported cases with the cervical, frontal, temporal, eyelids, and preauricular regions being the most frequent locations [3]. A female predominance has been reported with a male : female ratio of 2 : 3, and the vast majority of patients in the literature are Caucasian [3]. There has been association between multiple lesions with Gardner syndrome, myotonic dystrophy, and Turner's syndrome [9, 10].

Pilomatrixoma usually presents as an asymptomatic nodular single mass. The skin overlying the lesion is usually normal or may have reddish or bluish discoloration. These lesions are usually well circumscribed, spherical, or ovoid, and sometimes encapsulated. The behavior of pilomatrix carcinoma resembles that of basal cell carcinoma, but with the potential for metastasis. According to Bremnes et al., only 55 cases of pilomatrix carcinoma have been reported in the literature [8]. Rare cases of malignant pilomatrixoma with distant metastasis have been reported [11]. 

The diagnosis is usually established based on an incisional biopsy. However, fine needle aspiration cytology (FNAC) has been described as a preoperative diagnostic tool, but the results can be misleading and, at times, can lead to the erroneous diagnosis of a malignant neoplasm [12]. These tumors do not regress spontaneously and they require surgical excision. The surgeon should focus on preservation of the facial nerve branches that underlie the tumor without compromising adequate excision of the tumor. Few authors advised for a wide safety margin of 2 cm but this may be excessive given the rarity of malignant transformation of these tumors [13, 14]. Although this case report demonstrates multiple recurrences, pilomatrixoma has generally low recurrence rate (0–3%) [13–16]. This may be explained by inadequate conservative excision of previous lesions due to its sensitive location. Reconstruction is best achieved using local flaps because its good color match and simplicity. 

In summary, pilomatrixoma is a benign skin neoplasm that usually presents as a single, slow-growing subcutaneous or intradermal nodule. It is usually less than 3 cm and commonly affects the face. Giant pilomatrixoma (>5 cm), as in the presented case, is unusual. Malignant transformation has been reported but it is very rare. The diagnosis is established based on an incisional biopsy with questionable value of FNAC. The treatment of choice is surgical excision with a clear margin with every effort to preserve the branches of the facial nerve. Recurrence with adequate treatment is low.
